# Preoperative tissue Doppler imaging-derived atrial conduction time predicts postoperative atrial fibrillation in patients undergoing mitral valve surgery for mitral valve regurgitation

**DOI:** 10.1186/1749-8090-10-S1-A199

**Published:** 2015-12-16

**Authors:** Shinya Takahashi, Keijiro Katayama, Taijiro Sueda

**Affiliations:** 1 Department of Cardiovascular Surgery, Hiroshima University Hospital, Hiroshima, 7348551, Japan

## Background/Introduction

Postoperative atrial fibrillation (POAF) is a common complication of cardiac surgery and may result in stroke, heart failure and poor prognosis.

## Aims/Objectives

This study aimed to evaluate a novel index of total atrial conduction time derived from the P-wave onset (lead II) to the peak A'-wave on tissue Doppler imaging (PA-TDI duration). The PA-TDI duration was compared with previously reported predictors of POAF, and the optimal cut-off value of PA-DTI was calculated in patients undergoing mitral valve replacement or repair (MVR) for mitral valve regurgitation (MR).

## Method

Seventy-two patients undergoing MVR were enrolled. They had transthoracic echocardiography with tissue Doppler imaging preoperatively and were monitored postoperatively with continuous electrocardiographic telemetry for 14 days. Preoperative characteristics, echocardiographic data, operative data and postoperative findings were compared between patients with POAF (44 patients) and without (28 patients).

## Results

Postoperative cardiac and cerebral events was significantly larger in 44 patients with POAF than in 28 without POAF (14 patients (32%) vs. 2 (7%), p = 0.0190). Multivariate analysis revealed that etiology of degenerative (odds ratio [OR], 4.28; 95% confidence interval [CI], 1.30-14.10; p = 0.0169) and PA-TDI duration (OR, 1.04; CI, 1.01-1.07; p = 0.0052) were significant independent predictors of POAF. Receiver-operating-characteristic curve analysis showed the optimal cut-off values of PA-TDI duration was 159.4 ms.

## Discussion/Conclusion

Conclusions The PA-TDI duration was an independent predictor of POAF after MVR. Patients with PA-TDI duration >159.4 ms should be considered high risk and treated appropriately to improve outcomes.

